# Glucosinolates From Cruciferous Vegetables and Their Potential Role in Chronic Disease: Investigating the Preclinical and Clinical Evidence

**DOI:** 10.3389/fphar.2021.767975

**Published:** 2021-10-26

**Authors:** Emma L. Connolly, Marc Sim, Nikolaj Travica, Wolfgang Marx, Gemma Beasy, Gordon S. Lynch, Catherine P. Bondonno, Joshua R. Lewis, Jonathan M. Hodgson, Lauren C. Blekkenhorst

**Affiliations:** ^1^ Institute for Nutrition Research, School of Medical and Health Sciences, Edith Cowan University, Perth, WA, Australia; ^2^ Medical School, Royal Perth Hospital Research Foundation, The University of Western Australia, Perth, WA, Australia; ^3^ IMPACT—The Institute for Mental and Physical Health and Clinical Translation, School of Medicine, Barwon Health, Deakin University, Geelong, VIC, Australia; ^4^ Quadram Institute Bioscience, Norwich, United Kingdom; ^5^ Department of Anatomy and Physiology, Centre for Muscle Research, School of Biomedical Sciences, The University of Melbourne, Melbourne, VIC, Australia; ^6^ Centre for Kidney Research, Children’s Hospital at Westmead, School of Public Health, Sydney Medical School, The University of Sydney, Sydney, NSW, Australia

**Keywords:** glucosinolates, isothiocyanates, cardiometabolic disorders, neurological disorders, musculoskeletal health, cancer, cruciferous vegetables

## Abstract

An increasing body of evidence highlights the strong potential for a diet rich in fruit and vegetables to delay, and often prevent, the onset of chronic diseases, including cardiometabolic, neurological, and musculoskeletal conditions, and certain cancers. A possible protective component, glucosinolates, which are phytochemicals found almost exclusively in cruciferous vegetables, have been identified from preclinical and clinical studies. Current research suggests that glucosinolates (and isothiocyanates) act *via* several mechanisms, ultimately exhibiting anti-inflammatory, antioxidant, and chemo-protective effects. This review summarizes the current knowledge surrounding cruciferous vegetables and their glucosinolates in relation to the specified health conditions. Although there is evidence that consumption of a high glucosinolate diet is linked with reduced incidence of chronic diseases, future large-scale placebo-controlled human trials including standardized glucosinolate supplements are needed.

## Introduction

A poor diet with low amounts of fruits, vegetables, whole grains, seeds, and nuts, and excessive consumption of foods such as ultra-processed grains and sugar-sweetened beverages is the leading contributor to chronic disease risk (Afshin et al., 2019). There is increasing evidence that higher consumption of fruit and vegetables plays a central role in the prevention of non-communicable diseases (Liu, 2013). Vegetables contain a variety of different compounds that have beneficial health effects, including fiber, vitamins, minerals, and phytochemicals.

Research has shown that some types of vegetables may have greater health benefits than others for certain health conditions, such as diabetes, cardiovascular disease, and cancer (Cooper et al., 2012; Aune et al., 2017). This may be due to the presence of unique bioactive phytochemicals with potent health-related effects. Therefore, targeted recommendations surrounding the intake of specific types of vegetables with protective health benefits could assist in the reduction of non-communicable diseases.

Cruciferous vegetables include arugula (rocket), bok choy, broccoli, Brussels sprouts, cabbage, cauliflower, collard greens, daikon, horseradish, kale, kohlrabi, radish, turnips, wasabi, and watercress and are commonly consumed globally (International Agency for Research on Cancer, 2004). A number of epidemiological studies have investigated the health impact of cruciferous vegetables in humans and indicated that higher intakes of these vegetables are associated with a reduced risk of cardiometabolic diseases, musculoskeletal conditions, and cancer (Zhang et al., 2011; Liu et al., 2012; Wang et al., 2016; Aune et al., 2017; Blekkenhorst et al., 2017a; Sim et al., 2018). Although these vegetables contain a range of nutrients known to have beneficial health properties, studies have focused on the health effects of glucosinolates that are found almost exclusively in cruciferous vegetables.

**FIGURE 1:**
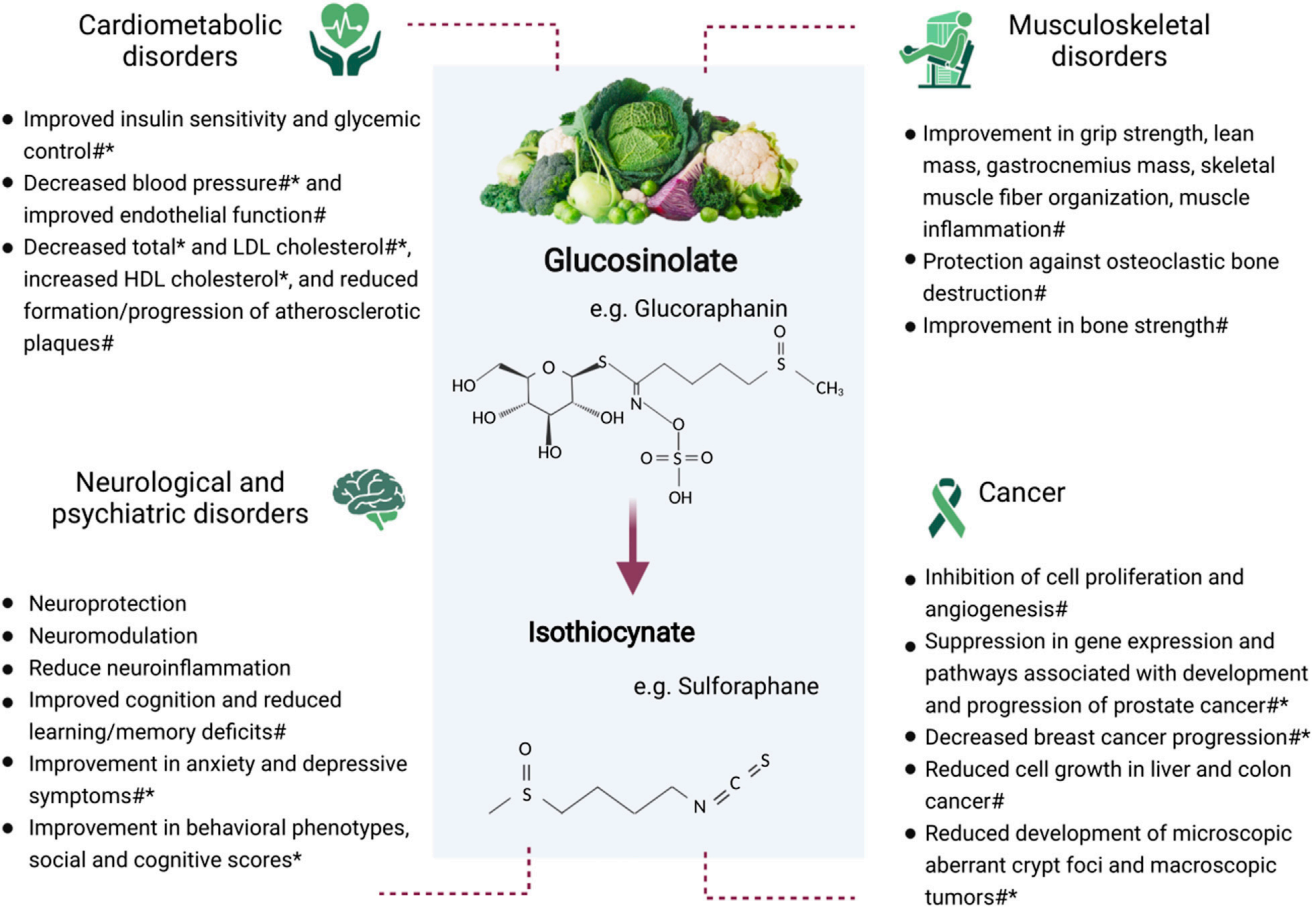
Potential benefits of glucosinolate consumption on cardiometabolic, neurological and psychiatric, and musculoskeletal conditions, and cancer. *Human study #Animal study.

Glucosinolates are phytochemicals that are proposed to be an important contributor to the health benefits of these vegetables (Manchali et al., 2012; Miękus et al., 2020). Glucosinolates (and their isothiocyanates) found in commonly consumed cruciferous vegetables include glucoraphanin (sulforaphane), sinigrin (allyl isothiocyanate), glucobrassicin, glucoraphasatin, and glucoiberin (Favela-González et al., 2020). Historically, a major research focus has been the anticancer effect of glucosinolates. However, there has been increasing evidence in recent years for the impact of cruciferous vegetables in cardiometabolic, neurological, and musculoskeletal conditions (Figure 1). Animal studies are important in identifying possible mechanisms responsible for health outcomes and provide the groundwork for future human trials. There is currently a lack of randomized controlled trials in this area, highlighting the importance of using animal data to inform trials investigating similar outcomes in humans. Therefore, the aim of this review was to provide an update of the current body of knowledge concerning the health effects of cruciferous vegetables and their glucosinolates, with a focus on cardiometabolic, neurological, and musculoskeletal conditions, and certain cancers. Electronic databases (PubMed, MEDLINE, Embase, and Google Scholar) were searched for peer-reviewed articles concerning glucosinolates and the specified health outcomes. The search was inclusive of all study designs given that this is an emerging area of research. Search terms included glucosinolates, isothiocyanates, cruciferous vegetables, cardiometabolic, hypertension, hyperglycemia, diabetes, dyslipidemia, neurological, psychiatric, musculoskeletal, muscle, bone, and cancer. Each subsection provides an overview of the epidemiological, preclinical, and clinical evidence.

## Glucosinolates in Cruciferous Vegetables

Cruciferous vegetables belong to the family *Brassicaceae* (also known as *Cruciferae*) within the order Brassicales. Within this order, almost all plants contain secondary plant metabolites known as glucosinolates, with commonly consumed plants belonging to the *Brassicaceae, Capparaceae,* and *Caricaceae* families (Shakour et al., 2021). Glucosinolates are responsible for the bitter taste and pungent odor found in these vegetables (Barba et al., 2016).

The type of glucosinolate determines the compounds formed; different sidechains result in the production of different end products (Barba et al., 2016). More than 130 glucosinolates have been identified (Blažević et al., 2020), although not all are found in plants commonly consumed by humans. Glucosinolates have been commonly classified under three categories: Aliphatic, indole, and aromatic glucosinolates. However, this classification has also been disputed in a recent review where the type of degradation end product was proposed to be a more useful classification method (Blažević et al., 2020).

Upon damage to plant tissue (i.e., by chewing, cutting, or mixing), the hydrolysis of glucosinolates *via* enzymatic activity of myrosinases occurs due to cellular breakdown. This results in the formation of products, including isothiocyanates, nitriles, and thiocyanates (Kissen et al., 2009). Metabolism of glucosinolates can also occur by gut microbiota (Luang-In et al., 2014). If myrosinase is denatured, when ingested, glucosinolates could be partially absorbed in the stomach and the remaining intact glucosinolates transit to the small intestine and colon where they may be hydrolyzed by intestinal microbiota and absorbed as isothiocyanates (Barba et al., 2016).

The glucosinolate profile of these vegetables is an important determinant of the ultimate biological action when consumed by humans, with the beneficial health properties of these compounds largely linked to the actions of isothiocyanates (Maina et al., 2020; Shakour et al., 2021). A number of factors influence the type and concentration of glucosinolates found in these vegetables, such as genotype, cultivar, cultivation site, growth conditions (e.g., temperature, nutrient availability, water content), plant stage, plant tissue analyzed, storage conditions, and preparation and cooking methods (Maina et al., 2020).

The plant genotype determines the specific glucosinolates that are present in a particular type of cruciferous vegetable. However, the interaction between these plant genetic factors and the environment can affect the concentration of glucosinolates found within a specific sample (Björkman et al., 2011; Raiola et al., 2017). Environmental factors include sulfate and nitrogen content of the soil, water availability or drought, and seasonality (Björkman et al., 2011). Furthermore, the developmental stage and specific plant tissue also influences the concentration of glucosinolates. For example, it has been found that 3-day old broccoli and cauliflower sprouts contain 10–100 times higher glucoraphanin levels per gram compared to their mature plant forms (Fahey et al., 1997).

Seasonal variation in glucosinolate content has been recorded, with the majority of studies indicating that plants with the highest glucosinolate concentration are typically grown in spring in intermediate temperatures with high light intensity, longer days, and dry conditions (Björkman et al., 2011). However, there is variation among plants and exceptions to this. The diurnal cycle may also impact the glucosinolate profile of cruciferous vegetables. For example, Casajús and coworkers found that glucosinolate levels were optimized during postharvest storage if broccoli were harvested close to noon (Casajús et al., 2020).

Typically, cruciferous vegetables are not consumed immediately after harvesting. Therefore, the storage and processing of such vegetables have a significant impact on glucosinolate content and the health benefit of consumption (Björkman et al., 2011; Barba et al., 2016). This is variable depending on the cultivar, plant tissue, and growth stage of the plant (Galgano et al., 2007). Freezing has been shown to result in higher retention of glucosinolates compared to refrigeration. Storage of broccoli at 6°C for 35 days resulted in a sulforaphane loss of 29%, compared to losses of approximately 13% after freezing at −18°C for 60 days. Moreover, the reduction in glucosinolates measured after freezing was associated with steam-blanching prior to freezing (Galgano et al., 2007). Storage in darkness has been shown to decrease the content of aliphatic glucosinolates (Casajús et al., 2020).

Cooking denatures the myrosinase found in cruciferous vegetables, with high temperature (>80°C) and long cooking time increasing the intensity of denaturation (Barba et al., 2016; Raiola et al., 2017). Therefore, steaming is the preferable cooking method to maximize glucosinolate yield compared to boiling, microwaving, and pressure cooking (Shakour et al., 2021). These other methods are all associated with significant glucosinolate losses up to more than 90% (Barba et al., 2016; Wang Z. et al., 2020; Shakour et al., 2021).

## Glucosinolates From Cruciferous Vegetables and Human Health

Many studies on the health effects of glucosinolates found in cruciferous vegetables have been based on laboratory or animal models. However, the number of studies involving humans is increasing. This review will focus on cardiometabolic, neurological, and musculoskeletal disorders in addition to providing a summary of the most recent literature on the effect of glucosinolates in certain cancers. Figure 1 provides an overview of the potential impact of glucosinolates on these conditions.

### Cardiometabolic Disorders

Cardiometabolic disorders are a collection of interrelated conditions, mainly dyslipidemia, insulin resistance, impaired glucose tolerance, hypertension, and central adiposity (Kirk and Klein, 2009). To date, investigations into the effects of cruciferous vegetables and their glucosinolates have largely focused on hyperglycemia and diabetes, hypertension, and dyslipidemia. Epidemiological studies have indicated a potential beneficial association between cruciferous vegetables and such conditions (Zhang et al., 2011; Blekkenhorst et al., 2017a; Chen et al., 2018; Lapuente et al., 2019; Zurbau et al., 2020). The antioxidant and anti-inflammatory properties of glucosinolates have been proposed to account for some of the observed health benefits associated with cruciferous vegetable intake and cardiometabolic disorders (Esteve, 2020). The anti-inflammatory effects of sulforaphane and other isothiocyanates for these conditions may involve increased Nrf2 activity and inhibition of NF-κB (Esteve, 2020).

#### Hyperglycemia and Diabetes

There is increasing interest in the impact of glucosinolates on glycemic control. Isothiocyanates, such as raphasatin and sulforaphane, may prevent or reduce glycemic-related complications in animal and human studies (Maina et al., 2020). Sulforaphane was found to prevent diabetes-induced hypertension and cardiac dysfunction in a study including hyperglycemic mice treated with or without sulforaphane (0.5 mg/kg daily for 5 days/week) for 3 months and with 3 months of further observation (Bai et al., 2013).

A number of observational studies have investigated associations between cruciferous vegetable intake and glucose metabolism and risk of type 2 diabetes. A meta-analysis including 11 prospective studies (754,729 participants, 58,297 incident type 2 diabetes cases) found a 13% lower risk of type 2 diabetes with high cruciferous vegetable intake (Chen et al., 2018). Unlike other studies, in a large prospective cohort study including 200,907 adults from the United States, self-reported total cruciferous vegetable consumption was significantly associated with the development of type 2 diabetes (HR: 1.16; 95% CI: 1.07, 1.25; P_trend_: < 0.001) (Ma et al., 2018). Participants in the highest quintile of glucosinolate intake had a 19% higher risk of type 2 diabetes than those in the lowest intake quintile (Ma et al., 2018). This differing outcome may be a result of other differences in diet, as the meta-analysis included European and Asian populations (Chen et al., 2018) in addition to studies from the United States (Ma et al., 2018). Differences in other foods or beverages consumed with cruciferous vegetables as part of the diet may differ greatly between these populations. However, potential faults in the measurement of glucosinolates in the study by Ma and coworkers have been noted (Oliviero et al., 2018), which may have also affected the results (e.g., the effect of vegetable processing and preparation was not considered in the food frequency questionnaire and use of total glucosinolates instead of the bioactive isothiocyanates).

Several human intervention trials have also been performed. In a 4-week parallel, randomized, double-blind placebo-controlled study including 81 human participants with type 2 diabetes, 10 g/day broccoli sprout powder (225 μmol sulforaphane daily) decreased fasting serum insulin and insulin resistance by 18.2 and 14.2%, respectively (Bahadoran et al., 2012). Positive results were also seen in a randomized double-blind placebo-controlled study including 97 Scandinavian patients with type 2 diabetes. In this study, patients consumed broccoli sprout extract (150 μmol sulforaphane/day) or a placebo over a 12-week period. The glucoraphanin-rich broccoli sprout extract improved both fasting glucose and HbA1c (7.38–7.04%) in obese patients with dysregulated diabetes (BMI >30 kg/m^2^; HbA1c > 50 mmol/mol) (Axelsson et al., 2017). The authors noted that this reduction in HbA1c was likely to reflect a clinically meaningful effect, as an HbA1c of 7% is the treatment goal of the American Diabetes Association (Axelsson et al., 2017). In a recent 12-week randomized controlled parallel intervention trial, 92 patients with type 2 diabetes were randomized to one of three different diets: 500 g/day bitter and strong-tasting root vegetables and cabbages, 500 g/day mild and sweet-tasting root vegetables and cabbages, or 120 g/day mild and sweet-tasting root vegetables and cabbages (normal diet) (Thorup et al., 2021). Improvements in glycemic control were noted in both groups with increased vegetable intake; however, consumption of bitter and strong-tasting vegetables (including kale and cabbage) had the greatest improvements to fasting glucose (4-fold), total cholesterol (2-fold), and body fat mass (2-fold) (Thorup et al., 2021).

#### Hypertension

Positive effects of glucosinolates in relation to blood pressure have also been found in animal models (Wu et al., 2004). Dried broccoli sprouts (200 mg/day; ∼12 μmol glucoraphanin/g dry weight) administered daily to spontaneously hypertensive stroke-prone (SHRSP) rats resulted in significantly decreased measures of oxidative stress [increased glutathione (GSH) and decreased oxidized glutathione (GSSG); GSH/GSSG ratio 10.6–14.0 compared with 4.0–7.2 with SHRSP rats on control diet], which was correlated with significantly lower blood pressure (20 mmHg lower compared to control) and improved endothelial function (Wu et al., 2004). Sulforaphane, the metabolite of glucoraphanin, was considered responsible for these changes. 10 μmol/kg body weight sulforaphane administered to SHRSP rats over a 4-month period resulted in reduced blood pressure and prevented hypertension-associated vascular remodeling (Senanayake et al., 2012). It has also been hypothesized that erucin, an analog of sulforaphane, may play a role in the antihypertensive effect seen with arugula (*Eruca sativa*) (Salma et al., 2018). A significant reduction in mean arterial pressure was reported in salt-induced hypertensive rats administered 10 and 30 mg/kg *E. sativa* extract (40.33 ± 1.15 and 59.43 ± 0.77% mmHg, respectively) (Salma et al., 2018).

Eighty-six adults with type 2 diabetes and a positive *H. pylori* stool antigen test were included in a 4-week study and randomized to one of three groups: standard triple therapy (twice daily 20 mg omeprazole, 500 mg clarithromycin, 1,000 mg amoxicillin for 14 days), broccoli sprout powder (6 g/day), or a combination of broccoli sprout powder and standard triple therapy. Whilst there was a reduction in systolic and diastolic blood pressure in all groups, this was only significant from baseline in the group who received the standard triple therapy and broccoli sprout powder combination (14 and 9.4 mmHg reduction in systolic and diastolic blood pressure, respectively) (Mirmiran et al., 2014). In another 4-week study including 40 individuals with hypertension found that daily ingestion of 10 g dried broccoli sprouts did not improve endothelial function (Christiansen et al., 2010). Conversely, although statistically non-significant (*p* = 0.05), a trend towards a ∼10% decrease in diastolic blood pressure was reported in pregnant women with preeclampsia during a recent dose escalation study of activated broccoli extract (equivalent to 32 or 64 mg sulforaphane), regardless of sulforaphane dose (Langston-Cox et al., 2021). A current randomized controlled crossover trial is investigating whether regular consumption of cruciferous vegetables results in short-term improvement in cardiometabolic measures, such as ambulatory blood pressure and glycemic control (Connolly et al., 2020).

#### Dyslipidemia

Most of the research investigating the effects of glucosinolate consumption on lipids has been undertaken in animal models. The sprout extract of Tuscan black cabbage, a type of kale, was found to be protective against a high-fat diet in rats, restoring antioxidant and phase II enzyme levels and lowering serum lipids (total cholesterol, triacylglycerides, non-esterified fatty acids) (Melega et al., 2013). Similarly, glucosinolates may contribute to the anti-inflammatory and cholesterol-lowering effects of red cabbage. Red cabbage microgreens (equivalent to 200 g vegetables/day/person) lowered circulating LDL cholesterol, liver cholesterol, and inflammatory cytokines in a rodent model of a high fat diet (Huang et al., 2016). A systematic review and meta-analysis of rodent models found consistent significant decreases in total serum cholesterol with sulforaphane doses of more than 0.5 mg/kg/day, and a reduction in LDL cholesterol has also been identified (Du et al., 2021). Glucosinolates have also been demonstrated to reduce the formation and progression of atherosclerotic lesions in rabbit models of atherosclerosis. Rabbits fed a high cholesterol diet and 0.25 mg/kg/day sulforaphane over 4-week had improved measures of endothelial function and resistance to the development of atherosclerosis, compared to rabbits fed only a high cholesterol diet (Shehatou and Suddek, 2016). Additionally, phenethyl isothiocyanate, the product of the glucosinolate gluconasturtiin, was shown to lower atherosclerotic plaque formation and hepatic lipid accumulation in C57BL/6 mice fed a high fat/cholesterol diet with 30 or 75 mg/kg/day phenethyl isothiocyanate (Gwon et al., 2020).

Few studies have been conducted in humans. In a phase I study conducted in Japan including 12 human participants (20–36 years), daily consumption of 100 g fresh broccoli sprouts was shown to improve HDL by 7.6% in female participants and reduce total cholesterol by 10% in male participants from baseline measurements (Murashima et al., 2004). Similarly, glucoraphanin-rich broccoli (400 g/week) consumed over a 12-week period was found to significantly reduce plasma LDL-C compared to consumption of standard broccoli in two randomized, double-blind parallel studies including 130 adults aged ≥50 years at risk of cardiovascular disease (Armah et al., 2015).

### Neurological and Psychiatric Disorders

Preclinical evidence suggests that glucosinolates and their metabolites, particularly sulforaphane, exhibit several biological properties that may be relevant to neurological and psychiatric conditions (Wu et al., 2016; Panjwani et al., 2018). A number of potential mechanisms include the modulation of the hypothalamic-pituitary-adrenal axis, oxidative stress, and inflammatory pathways. Sulforaphane may diminish neuroinflammation (by reducing NF-κB and TNF-*α*, and increasing IL-10) (Folkard et al., 2014; Santín-Márquez et al., 2019), reduce beta-amyloid and tau production (Kim, 2021), increase brain derived neurotrophic factor (Kim et al., 2017), postsynaptic density protein 95, AMPA receptor 1 (GluA1), dendritic spine density (Zhang et al., 2017), and blood brain barrier integrity (Li et al., 2013). Recently, sulforaphane has revealed epigenetic properties by inhibiting DNA methyltransferases in addition to preserving proteome homeostasis, influencing increased cellular lifespan and neurodegenerative prevention (Santín-Márquez et al., 2019). Furthermore, a sulforaphane intervention study in a cohort of healthy participants augmented peripheral and brain glutathione (Sedlak et al., 2017), an antioxidant readily implicated in various neurological functions (Dwivedi et al., 2020). At present, it remains unclear whether there are numerous key mechanisms involved across various disorders or whether mechanisms are disorder specific.

#### Psychiatric Disorders

Due to the supportive mechanistic data, animal models and clinical trials have begun to evaluate the use of sulforaphane interventions for psychiatric outcomes, including depression, schizophrenia, and autism (Panjwani et al., 2018). Acute and chronic consumption of sulforaphane consumption ameliorated anxiety and depressive-like behaviours in ICR and C57BL/6 mouse models (assessed using the novelty suppressed feeding test, open field and tail suspension tests) (Wu et al., 2016; Yao et al., 2016). Notably, C57BL/6 male mice that received sulforaphane in conjunction with depression-inducing lipopolysaccharide, exhibited lower depressive behaviours on the tail-suspension test and forced swimming test compared to those that did not receive sulforaphane (Zhang et al., 2017).

These results are supported by a recent randomized placebo controlled trial, which demonstrated that a 6-week sulforaphane intervention (30 mg/day) safely improved depressive symptoms, as measured by the Hamilton Rating Scale for Depression, in 66 participants with a history of a cardiac procedure and presence of mild to moderate depression (Ghazizadeh-Hashemi et al., 2021). In addition, an ongoing randomized controlled trial is examining whether sulforaphane may be utilized as an adjuvant treatment in bipolar depressive disorder (Wu et al., 2020). A small (*n* = 10) open label study demonstrated that an 8-week sulforaphane intervention improved cognitive function in a cohort of participants with schizophrenia (Shiina et al., 2015). Conversely, a more recent 16-week randomized controlled trial (*n* = 58) reported that sulforaphane (approximately 45 mg/day) failed to change the severity of psychotic symptoms compared to placebo using the total Positive and Negative Syndrome Scale (Dickerson et al., 2021). A recent review identified three double blind, randomized placebo controlled trials and two open-label trials examining the effects of sulforaphane on symptoms associated with Autism spectrum disorder (McGuinness and Kim, 2020). Collectively, study results were suggestive of a significant improvement in behavioral phenotypes such as irritability and motivation, alongside social and cognitive scores during sulforaphane interventions (Singh et al., 2014; Bent et al., 2018).

#### Neurological Disorders

Similar to psychiatry, various animal models have suggested the efficacy of sulforaphane interventions in the treatment of neurodegenerative disease; however, clinical trials are currently lacking (Schepici et al., 2020; Kim, 2021). Various pre-clinical animal studies have demonstrated improved cognitive ability and reduced learning/memory deficits following sulforaphane interventions in Alzheimer’s disease models (Kim et al., 2013; Lee et al., 2018). Further, administration of sulforaphane in adult mice induced a variety of cognitive changes associated with memory consolidation and spatial learning (Sunkaria et al., 2018). *In vitro* investigations have highlighted the potential of sulforaphane to prevent the loss of oligodendrocytes and axonal damage in multiple sclerosis, by modulating neuroinflammation and oxidative stress (Lim et al., 2016). Sulforaphane administration in mice exposed to 6-hydroxydopamine has also been shown to improve motor deficits, while dietary intake of sulforaphane preserved dopaminergic neurons following induced dopaminergic neurotoxicity in mice (Morroni et al., 2013; Pu et al., 2019). The protective effects of sulforaphane have further been implicated in acute neurodegenerative conditions, such as acute ischemic brain injury and traumatic brain injury, amongst mice models (Tarozzi et al., 2013). Given the promising results in animal studies, clinical studies are required to evaluate the effect of sulforaphane on neurological outcomes in humans.

### Musculoskeletal Disorders

Current epidemiological evidence suggests that cruciferous vegetables play a key role in muscle and bone health, possibly due to the presence of glucosinolates and isothiocyanates. For example, in a large cohort of older community-dwelling women (*n* = 1,468, ≥70 years), women with the highest cruciferous (>44 g/day) and allium (>11 g/day) vegetable intake had 28 and 34% lower relative hazards for a fracture-related hospitalization over 14.5 years, respectively (Blekkenhorst et al., 2017b). Similarly, greater cruciferous and allium vegetable intake was associated with 22 and 26% lower relative hazard ratio for an injurious fall-related hospitalization over this time, respectively (Sim et al., 2018). Although not specifically examining the role of glucosinolates, these studies demonstrate the potential importance of a diet rich in glucosinolates for musculoskeletal health.

#### Muscle

When considering the role of glucosinolates for musculoskeletal health, sulforaphane has been studied most extensively in animal models. Historically, the understanding of the effects of glucosinolates on skeletal muscle comes from trials of their application in animal production settings, with results indicating that although growth performance may be affected, muscle had an improved antioxidant status and fatty acid profile (Drażbo et al., 2019; Skugor et al., 2019). When sulforaphane was provided to male Wistar rats for 3 days prior to performing exhaustive exercise, sulforaphane served as an indirect antioxidant in skeletal muscle, as measured by decreased tissue total antioxidant capacity in the vastus lateralis muscle. It was concluded that sulforaphane plays a vital role in modulating the muscle redox environment; ultimately creating favorable effects on muscle (Malaguti et al., 2009). It was reported that type 2 diabetic mice receiving sulforaphane injections over 4 week had greater grip strength, lean mass, and gastrocnemius mass (Wang M. et al., 2020). These diabetic mice also presented with improved skeletal muscle fiber organization after sulforaphane treatment. It was proposed that sulforaphane may downregulate the expression of inflammatory and apoptotic associated proteins. Sulforaphane was also suggested to play a role in regulating mRNA levels of anti-inflammatory and oxidative related genes. Collectively, it was concluded that sulforaphane treatment could be protective against skeletal muscle disease in mice with type 2 diabetes.

Similar favorable results have also been reported in muscular dystrophy mice models, where sulforaphane treatment alleviated muscle inflammation in dystrophin-deficient *mdx* mice (Sun et al., 2015b). Three studies (from the same research group) investigated administration of sulforaphane to *mdx* dystrophic mice, the most widely utilised murine model of Duchenne muscular dystrophy (Swiderski and Lynch, 2021). Although there was some variation between the studies, the overall conclusion was that sulforaphane improved aspects of the dystrophic phenotype (Sun CC. et al., 2015; Sun et al., 2015c; Sun et al., 2016). Oral administration of sulforaphane (2 mg/kg/day) to 4-week-old *mdx* mice increased muscle mass, force production, and running distance compared to untreated *mdx* mice. These effects were associated with decreased expression of markers of muscle inflammation and fibrosis (Sun CC. et al., 2015; Sun et al., 2015c; Sun et al., 2016).

In the context of addressing aspects of sarcopenia or age-related impairments in skeletal muscle, 21-22-month-old mice were administered either a regular chow diet or the same diet supplemented with sulforaphane for 12 week (Bose et al., 2020). Skeletal muscle and heart function, mitochondrial function, and Nrf2 activity were assessed at the end of sulforaphane treatment. Compared to a young group of 2-month-old control mice, aged mice showed impairments in skeletal muscle and cardiac function and a decrease in Nrf2 activity. These parameters were all restored in the old mice receiving sulforaphane treatment. It was concluded that sulforaphane could be a safe and effective approach to protect against age-related impairments in skeletal muscle and the heart (Bose et al., 2020).

#### Bone

Sulforaphane has also been proposed to protect against osteoclastic bone destruction *in vitro* (Luo et al., 2021). Sulforaphane can support osteoblast differentiation (*via* epigenetic mechanisms) and expression of the osteoclast activator receptor activator of nuclear factor-κB ligand (RANKL) in osteocytes (Thaler et al., 2016). Here, after sulforaphane was provided over 5 week, the aforementioned effects correlated to a 20% greater bone volume in both normal and ovariectomized mouse models. Of note, no shifts in bone mineral density distribution were recorded. Ultimately, this led the authors to suggest that sulforaphane should be studied further due to its potential to counteract osteoporosis. Sulforaphane has also been studied for its potential benefits in osteoarthritis due to its reported antioxidant and anti-inflammatory properties. When a synthetic form of sulforaphane was orally supplemented to mice over 3 months, osteoarthritis-related gait asymmetry was recorded in vehicle-treated STR/Ort mice but not in sulforaphane treated mice (Javaheri et al., 2017). Sulforaphane treated mice also presented with improvements in trabecular and cortical bone, key indicators for bone strength. Noteworthy, favorable effects on bone turnover markers, such as C-terminal crosslinking telopeptide of type-I collagen (CTX-I; bone resorption marker) and total procollagen type 1 N-terminal propeptide (P1NP-bone formation marker), were recorded.

### Cancer

Epidemiological studies provide evidence that a diet rich in broccoli reduces cancer risk and progression (Yagishita et al., 2019; Le et al., 2020). Much of this link has been associated with isothiocyanates, including sulforaphane (Miękus et al., 2020; Gu et al., 2021). Sulforaphane is commonly recognized for its antioxidant, anti-inflammatory, and chemo-preventive effects with numerous types of cancer, including prostate, breast, liver, and colon (Soundararajan and Kim, 2018; Livingstone et al., 2019; Mahn and Castillo, 2021).

#### Prostate Cancer

Sulforaphane has been indicated to have multiple roles in a variety of key metabolic pathways involved in prostate cancer development including inhibition of angiogenesis and cell proliferation and initiating apoptosis (Traka et al., 2014; Soundararajan and Kim, 2018; Livingstone et al., 2019). Treatment with sulforaphane significantly reduced expression of key glycolytic genes, including hexokinase II and pyruvate kinase M2, in prostate cancer cell lines, LNCaP, 22RV1, and PC-3, and in transgenic mouse models, TRAMP and Hi-Myc (Singh et al., 2019). Sulforaphane also induced S-phase and G2/M-phase cell cycle arrest, enhanced histone acetylation, and up-regulated cell cycle proteins in the prostate cancer cell lines, DU145 and PC-3 (Rutz et al., 2020), suggesting that sulforaphane treatment leads to reduced cell proliferation activity.

There have been numerous human studies investigating the influence of sulforaphane on prostate cancer. Traka and coworkers evaluated the effect of a once-a-week consumption of broccoli soups with increasing concentration of glucoraphanin in a prostate cancer cohort on active surveillance. They indicated a dose-dependent suppression in gene expression and pathways associated with the development and progression of prostate cancer (Traka et al., 2019). Consistent with this study, Zhang and coworkers assessed the influence of daily consumption of either 200 µmol broccoli sprout extract or placebo in men scheduled for prostate biopsy and demonstrated changes in gene expression with the downregulation of genes associated with prostate cancer progression (*AMACR* and *ARLNC1*) (Zhang et al., 2020).

#### Breast Cancer

Previous cell-based and human studies have indicated that sulforaphane exposure is associated with decreased breast cancer progression, including initiating cell cycle arrest and apoptosis (Kuran et al., 2020). Sulforaphane treatment led to the inhibition of cell proliferation of breast cancer cells lines, MCF-7 and MDA-MB-231 and provoked cytotoxic activity by changing cysteine residues of the promyelocytic leukemia protein, known to promote proliferation of the MCF-7 breast cancer cell line (Alhazmi et al., 2020). Sulforaphane has also been shown to modify cell migration and expression of epithelial mesenchymal transition markers that play a large role in breast cancer metastasis (Bagheri et al., 2020).

There are limited human studies that have evaluated the role of sulforaphane on breast cancer. Atwell and coworkers evaluated the effect of either a supplement containing standardized concentration of glucoraphanin with myrosinase enzyme (BroccoMax) or placebo for a minimum of 2-week in women with abnormal mammograms and booked for breast biopsies. They indicated that BroccoMax supplementation significantly reduced the tissue biomarkers, Ki-67 and HDAC3, in benign tissue but not in ductal carcinoma *in situ* (DCIS) or invasive ductal carcinoma breast tissues (Atwell et al., 2015). Zhang and coworkers evaluated the interaction between cruciferous vegetable intake and biomarkers in women scheduled for breast biopsies and found a negative association with total cruciferous vegetable intake and cell proliferation in DCIS breast tissue (Zhang et al., 2016), suggesting a role of sulforaphane in chemoprevention against breast cancer.

#### Liver Cancer

Treatment with sulforaphane significantly reduced migration and adhesion and inhibited expression of key molecules in angiogenesis, VEGF, STAT3, and HIF-1*α*, in liver cancer cell line, HepG2 (Liu et al., 2017). Sulforaphane exposure led to reduction in cell growth, by significantly downregulating key cell cycle-related genes in HepG2 and Huh7 cell lines, and also within a xenograft mouse model (Sato et al., 2018). Sulforaphane decreased secretion of the pro-inflammatory cytokine, interleukin-6, and was not linked with cellular toxicity in HepG2 cells (Al-Bakheit and Abu-Qatouseh, 2020), suggesting that sulforaphane could have anti-inflammatory properties and may modulate liver carcinogenesis.

Whilst there is much cell-based evidence for the role of sulforaphane on liver cancer, there are limited animal and no human studies. Chen and coworkers assessed the effect on mice given diethylnitrosamine before being placed on a Western diet or Western diet supplemented with 10% w/w freeze-dried broccoli powder. The broccoli diet decreased liver damage and fatty liver progression but did not reduce liver cancer development (Chen et al., 2016a). However, in another study, mice were given a control or Western diet with or without the addition of 10% w/w freeze-dried broccoli before being treated with diethylnitrosamine. The initiation and progression of liver cancer was reduced in mice receiving the freeze-dried broccoli (Chen et al., 2016b). Thus, further animal and human studies are required to delineate the effect of sulforaphane on liver cancer.

#### Colon Cancer

Colon cancer incidence is heavily associated with dietary habits; diets rich in red meat are associated with an increased risk, whilst those high in fruit and vegetables are associated with a reduced risk (Veettil et al., 2021). In human colon cancer cell lines, HCT116 and SW480, sulforaphane diminished cell growth in a dose-dependent way, and increased apoptosis induced by *Lactobacillus* through the TNF*α* pathway (Yasuda et al., 2019). Sulforaphane treatment also led to suppression of key colorectal cancer stem cell markers, such as CD44 and CD133 in HCT116 and SW480 spheroids, facilitated by the TAp63*α*/Lgr5/*β*-catenin pathway (Chen et al., 2020), suggesting that exposure to sulforaphane could lead to lowered cell proliferation activity. On the contrary, sulforaphane treatment (<10 µM) led to increased cell proliferation and also lowered expression of key apoptotic proteins Bcl-2 and Bax, but concentrations of greater than 10 µM caused cell death in p53-wild-type HCT116 cells (Wang et al., 2021). There are few animal or human studies that have assessed the role of sulforaphane on colon cancer. However, Suzuki and coworkers found that sulforaphane intake led to reduced development of microscopic aberrant crypt foci and macroscopic tumors in mice or patients with colon cancer (Suzuki et al., 2019), suggesting that consumption of broccoli could prevent colon cancer progression.

## Conclusion and Future Directions

The evidence presented in this review suggests that glucosinolates and their isothiocyanate metabolites found in cruciferous vegetables are important components in the prevention and treatment of multiple chronic diseases. Studies indicate that cruciferous vegetables and their glucosinolates may have an impact on a number of cardiometabolic disorders. Of note, improvements in glycemic control, blood pressure, and lipid profile have been identified, which may lead to a reduction or delay in disease progression. Likewise, glucosinolate metabolites, particularly sulforaphane, may exert a beneficial effect on neurological and psychiatric conditions, such as depression, schizophrenia, autism, Alzheimer’s disease, and multiple sclerosis. Sulforaphane has also been indicated as an important dietary component for musculoskeletal disorders, with studies reporting improvements in measures for both muscle and bone. To date, however, most of the aforementioned results have come from animal models, with limited human randomized controlled trials. Although there are limitations in the extrapolation of animal data directly to humans, animal studies provide an important insight into the mechanisms by which glucosinolates may also exert outcomes in humans.

Moreover, cruciferous vegetables also contain a variety of other nutrients known to have important health effects (e.g., vitamin C, vitamin K, carotenoids, and flavonoids) (Manchali et al., 2012). As such, it is difficult to separate the action of glucosinolates from these other compounds when determining the results of food-based cruciferous vegetable interventions. Further, when these vegetables are consumed as part of a particular dietary pattern, there may be other factors influencing the effect of glucosinolates consumed (e.g., influences on metabolism and gut microbiota). This idea is also relevant to observational studies, where other diet components could influence the observed associations between glucosinolate-rich vegetables and particular health outcomes. Therefore, future large-scale human trials utilizing standardized, reproducible protocols, and appropriate glucosinolate doses that reflect realistic habitual intake are needed to further elucidate the benefits of cruciferous vegetables and related compounds.

Whilst there is also evidence that sulforaphane reduces cancer development, future research on the underlying molecular mechanisms is also warranted to further understand the role of sulforaphane in the metabolic rewiring of cancer progression. Interestingly, recent studies have indicated that sulforaphane treatment in conjunction with anti-cancer treatments such as chemotherapy, increases cancer cell sensitivity (Jabbarzadeh Kaboli et al., 2020), lessens their toxic side effects (Calcabrini et al., 2020), and inhibits key survival pathways in cancer progression (Mokhtari et al., 2021). This suggests that sulforaphane could not only be a potential drug candidate but also be used in combination with current anti-cancer treatments.

A number of different factors can influence the glucosinolate content of vegetables (e.g., cultivar, growth conditions, storage, and cooking method), and this variation in glucosinolate content may be partially responsible for differing results from studies utilizing the same vegetables (e.g., broccoli) across different locations and with varying preparation and cooking methods. Further studies are also needed to determine and utilize the optimal dose and delivery of sulforaphane for treating different aspects of the dystrophic pathology and for its potential future application as a nutraceutical for treating neuromuscular disorders. Additionally, future studies should examine whether sulforaphane could address different aspects of age-related impairments in skeletal muscle function, including injury susceptibility, fatigue, and muscle regenerative capacity.

Given the positive actions of glucosinolates are largely related to anti-inflammatory and antioxidant mechanisms, the consumption of cruciferous vegetables and their glucosinolates may also have beneficial outcomes on other health conditions. Although there are few studies investigating the impact of certain cruciferous vegetables on other chronic conditions, such as obesity, future studies are needed to further investigate the role of glucosinolates.
